# Combining network topology and information theory to construct representative brain networks

**DOI:** 10.1162/netn_a_00170

**Published:** 2021-02-01

**Authors:** Andrea I. Luppi, Emmanuel A. Stamatakis

**Affiliations:** Division of Anesthesia, School of Clinical Medicine, University of Cambridge, Cambridge, United Kingdom; Department of Clinical Neurosciences, University of Cambridge, Cambridge, United Kingdom; Division of Anesthesia, School of Clinical Medicine, University of Cambridge, Cambridge, United Kingdom; Department of Clinical Neurosciences, University of Cambridge, Cambridge, United Kingdom

**Keywords:** Graph theory, Parcellation, Thresholding, Representativeness, Functional connectivity, Structural connectivity

## Abstract

Network neuroscience employs graph theory to investigate the human brain as a complex network, and derive generalizable insights about the brain’s network properties. However, graph-theoretical results obtained from network construction pipelines that produce idiosyncratic networks may not generalize when alternative pipelines are employed. This issue is especially pressing because a wide variety of network construction pipelines have been employed in the human network neuroscience literature, making comparisons between studies problematic. Here, we investigate how to produce networks that are maximally representative of the broader set of brain networks obtained from the same neuroimaging data. We do so by minimizing an information-theoretic measure of divergence between network topologies, known as the portrait divergence. Based on functional and diffusion MRI data from the Human Connectome Project, we consider anatomical, functional, and multimodal parcellations at three different scales, and 48 distinct ways of defining network edges. We show that the highest representativeness can be obtained by using parcellations in the order of 200 regions and filtering functional networks based on efficiency-cost optimization—though suitable alternatives are also highlighted. Overall, we identify specific node definition and thresholding procedures that neuroscientists can follow in order to derive representative networks from their human neuroimaging data.

## INTRODUCTION

Network neuroscience has provided compelling evidence that viewing the human brain as a network can provide powerful insights into both healthy and pathological cognition (Bullmore & Sporns, 2009; Sporns, 2013; Sporns, Tononi, & Kötter, 2005). Mathematically, a network consists of elements (nodes) connected by edges. To turn their human neuroimaging data into networks, researchers therefore need to define the networks’ nodes and edges (Craddock et al., 2013).

Node definition is the problem of identifying meaningful units in the network: Multiple approaches have been proposed to parcellate the human brain into distinct regions, with varying levels of granularity and based on features such as anatomy, homogeneity, and structural and functional connectivity (Arslan et al., 2018). For MRI data, a recent review identified over 50 distinct methods (Hallquist & Hillary, 2019).

A further issue arises for functional connectivity (FC), which is typically given by some measure of statistical association between nodes’ time series (e.g., Pearson correlation, mutual information, phase coherence), as recorded by functional MRI or MEG/EEG (Craddock et al., 2013). Each node will have some degree of mathematical relationship with every other node, which may or may not be underpinned by a true neuronal basis: Since anatomical connectivity in the brain is known to be sparse (Sporns, 2011), at least some functional connections are likely to be false positives due to statistical noise (Rubinov & Sporns, 2010).

Therefore, researchers need to determine which connections to retain, and which ones to reject, a step known as “edge filtering” This can be based on arbitrary thresholds for edge strength or density, but an inappropriate choice could obscure biological differences of interest (van den Heuvel et al., 2017), or introduce confounds in subsequent graph-theoretical analyses (Garrison, Scheinost, Finn, Shen, & Todd Constable, 2015). Data-driven filtering methods have also been proposed, whether based on statistical considerations (Smith et al., 2011), or optimizing some objective criterion (De Vico Fallani, Latora, & Chavez, 2017; Dimitriadis, Antonakakis, Simos, Fletcher, & Papanicolaou, 2017).

Thus, there are a vast number of possible ways to turn neuroimaging data into brain networks; we refer to these as “network construction pipelines.” Crucially, just like outliers can make statistical generalizations unreliable, so brain network construction pipelines that produce idiosyncratic networks may yield graph-theoretical results that do not generalize well to the broader set of all possible brain networks constructed from the same neuroimaging data. This issue is especially pressing because a wide variety of network construction pipelines have been employed in the network neuroscience literature (Craddock et al., 2013; Hallquist & Hillary, 2019), making comparisons between studies and meta-analyses problematic.

Existing work comparing aspects of brain network construction has sought to rank pipelines based on reproducibility or test-retest reliability (Andellini, Cannatà, Gazzellini, Bernardi, & Napolitano, 2015; Arslan et al., 2018; Braun et al., 2012; B. Cao et al., 2019; H. Cao et al., 2014; Du et al., 2015; Hu et al., 2019; Messaritaki, Dimitriadis, & Jones, 2019; Termenon, Jaillard, Delon-Martin, & Achard, 2016; J.-H. Wang et al., 2011; J. Wang et al., 2017; Welton, Kent, Auer, & Dineen, 2015), or in terms of maximizing discriminative ability or informativeness of the resulting networks (Arslan et al., 2018; De Vico Fallani et al., 2017; Dimitriadis, Salis, Tarnanas, & Linden, 2017). However, to our knowledge a systematic evaluation of the representativeness of brain networks, in terms of similarity between networks obtained from different construction pipelines applied to the same brain data, has not been undertaken before.

To address this question and compare networks derived from different construction pipelines, we leverage a recently proposed measure of information-theoretic distance between the topologies of different networks, termed portrait divergence (PD; Bagrow & Bollt, 2019). Thanks to its information-theoretic underpinning, the portrait divergence may be interpreted as the information loss when using one network to represent another. Thus, the network with minimum average PD from all others will also be the one that can be used as a proxy for all others with the lowest information loss—which we take to mean that it is the most representative of the set of all networks under consideration (here, the networks produced by applying different construction pipelines to the same neuroimaging data).

Focusing on MRI, here we employ the portrait divergence to compare structural and functional brain networks obtained from nine different combinations of parcellation method (based on anatomical features, regional homogeneity, and multimodal) and number of nodes (approximately 100, 200, and 400). We seek to identify which combination produces the most representative network, defined as the network with the smallest average divergence from all others. We also introduce a new filtering scheme based on matching the density of functional and structural networks, termed structural density matching, and we investigate a total of 12 different filtering schemes to determine which one produces functional networks with the least divergence across parcellations and edge definitions.

## MATERIALS AND METHODS

### Human Connectome Project Data

#### HCP: Dataset description.

The dataset of functional and structural neuroimaging data used in this work came from the Human Connectome Project (HCP, http://www.humanconnectome.org/), Release Q3. Per HCP protocol, all subjects gave written informed consent to the HCP Consortium. These data contained fMRI and diffusion-weighted imaging (DWI) acquisitions from 100 unrelated subjects of the HCP 900 data release (Van Essen et al., 2013). All HCP scanning protocols were approved by the local Institutional Review Board at Washington University in St. Louis.

#### HCP: Functional data acquisition.

The following sequences were used: structural MRI: 3D MPRAGE T1-weighted, TR = 2,400 ms, TE = 2.14 ms, TI = 1,000 ms, flip angle = 8°, FOV = 224 × 224, voxel size = 0.7 mm isotropic. Two sessions of 15-min resting-state fMRI: gradient-echo EPI, TR = 720 ms, TE = 33.1 ms, flip angle = 52°, FOV = 208 × 180, voxel size = 2 mm isotropic. Here, we used functional data from only the first scanning session, in LR direction. HCP-minimally preprocessed images were used for all acquisitions (Glasser et al., 2013).

#### HCP: Diffusion-weighted data.

We used diffusion MRI (dMRI) data from the 100 unrelated subjects of the HCP 900-subject data release (Van Essen et al., 2013). The diffusion-weighted acquisition protocol is covered in detail elsewhere (Glasser et al., 2013). The diffusion MRI scan was conducted on a Siemens 3T Skyra scanner using a 2D spin-echo single-shot multiband EPI sequence with a multiband factor of 3 and monopolar gradient pulse. The spatial resolution was 1.25 mm isotropic. TR = 5,500 ms, TE = 89.50 ms. The b-values were 1,000, 2,000, and 3,000 s/mm^2^. The total number of diffusion sampling directions was 90, 90, and 90 for each of the shells in addition to six b0 images. We used the version of the data made available in DSI Studio–compatible format at http://brain.labsolver.org/diffusion-mri-templates/hcp-842-hcp-1021 (Yeh et al., 2018).

### Functional MRI Preprocessing and Denoising

We used the minimally preprocessed fMRI data from the HCP, which includes bias field correction, functional realignment, motion correction, and spatial normalization to Montreal Neurological Institute (MNI-152) standard space with 2 mm isotropic resampling resolution (Glasser et al., 2013). We also removed the first 10 volumes, to allow magnetization to reach steady state. Additional denoising steps were performed using the SPM12-based (http://www.fil.ion.ucl.ac.uk/spm) toolbox CONN (http://www.nitrc.org/projects/conn), version 17f (Whitfield-Gabrieli & Nieto-Castanon, 2012). To reduce noise due to cardiac and motion artifacts, we applied the anatomical CompCor method of denoising the functional data. The anatomical CompCor method (also implemented within the CONN toolbox) involves regressing out of the functional data the following confounding effects: the first five principal components attributable to each individual’s white matter signal, and the first five components attributable to individual cerebrospinal fluid (CSF) signal; and six subject-specific realignment parameters (three translations and three rotations) as well as their first-order temporal derivatives (Behzadi, Restom, Liau, & Liu, 2007). Linear detrending was also applied, and the subject-specific denoised BOLD signal time series were band-pass filtered to eliminate both low-frequency drift effects and high-frequency noise, thus retaining frequencies between 0.008 and 0.09 Hz.

The step of global signal regression (GSR) has received substantial attention in the literature, with inconclusive results about whether its effect on subsequent network construction is beneficial (Braun et al., 2012; Welton et al., 2015), deleterious (H. Cao et al., 2014), or null (Andellini et al., 2015; Du et al., 2015). Here, we chose to avoid GSR in favor of the aCompCor denoising procedure, because GSR mathematically mandates that approximately 50% of correlations between regions will be negative (Braun et al., 2012), but evidence indicates that the proportion of anticorrelations contains relevant biological information (Luppi et al., 2019).

### DWI Reconstruction and Fiber Tracking

The minimally preprocessed DWI HCP data (Glasser et al., 2013) were corrected for eddy current and susceptibility artifact. DWI data were then reconstructed using q-space diffeomorphic reconstruction (QSDR; Yeh, Wedeen, & Tseng, 2011), as implemented in DSI Studio (http://dsi-studio.labsolver.org). QSDR is a model-free method that calculates the orientational distribution of the density of diffusing water in a standard space, to conserve the diffusible spins and preserve the continuity of fiber geometry for fiber tracking. QSDR first reconstructs diffusion-weighted images in native space and computes the quantitative anisotropy (QA) in each voxel. These QA values are used to warp the brain to a template QA volume in MNI space using a nonlinear registration algorithm implemented in the statistical parametric mapping (SPM) software. A diffusion sampling length ratio of 2.5 was used, and the output resolution was 1 mm.

A modified FACT algorithm (Yeh, Verstynen, Wang, Fernández-Miranda, & Tseng, 2013) was then used to perform deterministic fiber tracking on the reconstructed data, with the following parameters (Medaglia et al., 2016). Angular cutoff of 55°, step size of 1.0 mm, minimum length of 10 mm, maximum length of 400 mm, spin density function smoothing of 0.0, and a QA threshold determined by DWI signal in the colony-stimulating factor. Each of the streamlines generated was automatically screened for its termination location. A white matter mask was created by applying DSI Studio’s default anisotropy threshold (0.6 Otsu’s threshold) to the spin distribution function’s anisotropy values. The mask was used to eliminate streamlines with premature termination in the white matter region. Deterministic fiber tracking was performed until 1,000,000 streamlines were reconstructed for each individual.

### Parcellations

The most common approach for defining nodes in brain networks is to aggregate multiple neighboring voxels into parcels, in order to obtain nodes with interpretable neurobiological meaning while also reducing the computational burden. This can be done based on anatomical boundaries and cytoarchitecture (Cammoun et al., 2012; Tzourio-Mazoyer et al., 2002), similarity of structural (Beckmann, Johansen-Berg, & Rushworth, 2009) or functional connectivity (Bellec et al., 2006; Schaefer et al., 2018), or by a variety of other means (Arslan et al., 2018; Cohen et al., 2008; Craddock, James, Holtzheimer, Hu, & Mayberg, 2012; Eickhoff, Yeo, & Genon, 2018; Fan et al., 2016; Glasser et al., 2016).

The parcellation size (granularity) constitutes a further consideration to the method used to determine how voxels are grouped together into parcels/nodes. It represents a trade-off between spatial detail, on the one hand, and robustness and interpretability on the other. Spatial averaging (by grouping together many neighboring voxels) can mitigate errors due to registration and MRI artifacts, and smaller parcels may be more susceptible to noise; however, finer-grained parcellations may detect subtle differences that would otherwise go unnoticed because of spatial averaging (Cammoun et al., 2012).

Thus, a large number of parcellations exist, using different criteria to aggregate voxels, and varying in size from a few tens to over 1,000 parcels (Arslan et al., 2018). To account for both parcellation method and granularity, here the preprocessed diffusion-weighted and functional MRI data were parcellated according to the following schemes (all in MNI volumetric standard space).

The Lausanne atlas is a multiscale anatomical parcellation obtained from progressively finer subdivisions of the 66 sulcus-based parcels of the Desikan-Killiany anatomical atlas (Cammoun et al., 2012). Here we included versions with 129, 234, and 463 cortical and subcortical nodes.

We also considered the recent cortical parcellation by Schaefer and colleagues (Schaefer et al., 2018), which integrates local gradient and global similarity approaches from task-based and resting-state functional connectivity. This is also a multiscale parcellation, and here we considered the scales with 100, 200, and 400 nodes, respectively. Since the Schaefer parcellation does not include subcortical regions, to make it comparable with the other parcellation schemes (and to give proper consideration to these important regions) we supplemented it with the recently developed Melbourne subcortical atlas (Tian, Margulies, Breakspear, & Zalesky, 2020). We chose this atlas because it is also based on resting-state and task-based functional connectivity, thus being consistent with the parcellation methodology of the Schaefer cortical atlas; and because it is also a multiscale atlas. Specifically, the 100-node Schaefer parcellation was supplemented with the 16-node version of the Melbourne atlas, yielding a total of 116 nodes; the 200-node Schaefer parcellation was supplemented with the 32-node version of the Melbourne atlas, yielding a total of 232 nodes; and the 400-node Schaefer parcellation was supplemented with the 54-node version of the Melbourne atlas, yielding a total of 454 nodes (thus, the total number of nodes approximately doubled each time, and was consistent with the number of nodes present in the Lausanne parcellation at corresponding scales).

Finally, we included three popular atlases based on different information, each of which comes at a single scale of resolution. The Automated Anatomical Labelling (AAL) atlas (Tzourio-Mazoyer et al., 2002) is one of the most widely used parcellations in network neuroscience (Hallquist & Hillary, 2019). It comprises 90 cortical and subcortical regions (the 26 cerebellar parcels were excluded), obtained by anatomical parcellation of the high-resolution T1 volume of the same subject scanned 27 times. The Brainnetome atlas is a set of 210 cortical and 36 subcortical regions, identified by combining multimodal anatomical and functional connectivity information, further informed by BrainMap data about regional functional profiles (Fan et al., 2016). Finally, the Glasser parcellation comprises 360 cortical regions identified by combining multimodal information about cortical architecture, function, connectivity, and topography, obtained from a large number of high-resolution HCP data (Glasser et al., 2016). The Glasser parcellation was also supplemented with the 54-region version of the Melbourne atlas, in order to include a comparable number of subcortical regions. When conflicts arose (i.e., the same voxel being assigned to one parcel of the Melbourne atlas, and one parcel of the Glasser atlas; primarily for hippocampal parcels), the Glasser atlas was given precedence in the assignment. No region was entirely overlapping between these two atlases, however, so the number of final nodes was 360 + 54 = 414.

Thus, the chosen atlases spanned three scales (approximately 100, 200, and 400 nodes) and three different ways of deriving parcels: based on anatomical considerations (Lausanne and AAL), based on functional connectivity (Schaefer), and informed by multimodal considerations (Brainnetome, Glasser). The parcellation schemes and sizes are summarized in Table 1.

**Table 1.** Atlases adopted in the present study, by scale (rows) and method (columns)

	**Anatomical**	**Functional**	**Mixed**
**Scale-100**	Lausanne-129	Schaefer-100 + Melbourne-16	AAL-90
**Scale-200**	Lausanne-234	Schaefer-200 + Melbourne-32	Brainnetome-246
**Scale-400**	Lausanne-463	Schaefer-400 + Melbourne-54	Glasser-360 + Melbourne-54

### Structural Connectivity

To construct matrices of structural connectivity between brain regions, every subject’s diffusion tensor imaging (DTI) data were parcellated according to each of the parcellation schemes considered here. Then, an undirected connectivity matrix S was derived by setting entry *S*_*ij*_ = log_10_(1 + *N*_*ij*>_), with *N*_*ij*_ being the number of white matter streamlines between regions *i* and *j*. The logarithm was used to reduce the skewness of the distribution of edge weights, with unity added to ensure that zero-valued entries would remain zero after the transformation. This resulted in one weighted matrix of structural connectivity per parcellation, per subject. Finally, the weights were normalized to lie between 0 and unity, by dividing each edge by the maximum value of the matrix.

### Human Structural Connectome Template

To obtain the most accurate estimate possible of the human structural connectome, which is required for the structural density matching filtering procedure (described below), we relied on the HCP group-average template constructed and made publicly available by Yeh and colleagues (Yeh et al., 2018). The HCP group-average template was constructed from a total of 1,021 subjects’ diffusion MRI data from the Human Connectome Project (2017 Q4, 1,200-subject release). The DWI acquisition parameters were the same as described earlier. The diffusion data were reconstructed in the MNI space using QSDR (Yeh et al., 2011) to obtain the spin distribution function (Yeh, Wedeen, & Tseng, 2010). A diffusion sampling length ratio of 2.5 was used, and the output resolution was 1 mm. The analysis was conducted using DSI Studio (http://dsi-studio.labsolver.org).

Based on this group-average connectome, the same fiber tracking and parcellation procedures were applied as for the individual DTI data. The density of the resulting connectivity matrix, for each parcellation, was then used to determine the density threshold for the structural density matching filtering (see below).

### Functional Connectivity

The subsequent steps were performed separately for each parcellation. The time courses of denoised BOLD signals were averaged between all voxels belonging to a given region of interest (ROI), and the resulting ROI-specific time courses of each subject were then extracted for further analysis in MATLAB version 2016a.

For each subject and parcellation, a matrix of functional connectivity *F* between each pair of ROIs was then estimated as follows: For each pair of nodes *i* and *j*, *F*_*ij*_ was given by the Pearson correlation coefficient between the time courses of *i* and *j*, over the full scanning length. Since Person correlation can yield negative as well as positive values, we followed common practice in network neuroscience and set all negative values in each FC matrix to 0. However, to ensure that our results were not biased by ignoring negative correlations, functional connectivity between nodes was also estimated using mutual information (MI), which does not produce negative-valued edges. Mutual information *I* quantifies the interdependence between two random variables *X* and *Y*. It corresponds to the average reduction in uncertainty about *X* when *Y* is given (or vice versa, since this quantity is symmetric):

I(X;Y)=H(X)+H(Y)−H(X,Y)=H(X)−H(X|Y), (1)

with *H*(*X*) being the Shannon entropy of a variable *X*. Thus, mutual information quantifies the information that a given variable provides about another, including both linear and nonlinear relationships. To ensure a comparable range of values between MI and (positive) Pearson correlation, the values in each individual matrix of MI-based functional connectivity were normalized to lie between 0 and unity, by dividing each edge by the maximum value in the matrix.

### Filtering Schemes

Functional connectivity (whether measured by Pearson correlation or mutual information) produces maximally dense matrices, in which each node has some degree of association with each other node. Since brain networks are known to have sparse anatomical connectivity (Sporns, 2011), a maximally dense network is biologically implausible, and many functional connections are likely to be spurious. Thus, some form of filtering is typically employed to obtain a sparse network of functional connectivity.

Here, we considered 12 different filtering approaches (Table 2). For each of the approaches described below, and each parcellation scheme, a given subject’s functional connectivity network was constructed by retaining only the edges selected by the filtering scheme, and setting all other edges to 0, resulting in a sparse, undirected graph.

**Table 2.** Filtering schemes adopted in the present study

**Filtering scheme**	**Description**
Fixed density 5% (FD-5%)	Top 5% of strongest edges
Fixed density 10% (FD-10%)	Top 10% of strongest edges
Fixed density 20% (FD-20%)	Top 20% of strongest edges
Fixed density 40% (FD-40%)	Top 40% of strongest edges
Absolute threshold 0.1 (Abs0.1)	Edges with value > 0.1
Absolute threshold 0.3 (Abs0.3)	Edges with value > 0.3
Absolute threshold 0.5 (Abs0.5)	Edges with value > 0.5
Efficiency cost optimization (ECO)	Average node degree = 3, to maximize trade-off between overall efficiency and wiring cost
Minimum spanning tree-ECO (MST-ECO)	Same as ECO, but including the network’s minimum spanning tree among the selected edges
Structural density matching (SDM)	Proportional thresholding, with same density as the HCP group-average DTI data parcellated using the same atlas
Orthogonal minimum spanning trees (OMST)	Optimization of global efficiency minus wiring cost, by combining independent minimum spanning trees of the network
**Random (Random20%)**	20% of edges chosen at random

#### Proportional thresholding.

While this is perhaps the most common approach in the network neuroscience literature, there is no gold standard for which proportion of the strongest edges to retain when constructing a network from functional connectivity data. We therefore employed four different density levels as threshold values, in the range of densities commonly adopted in the literature: fixed density (FD) of 5%, 10%, 20%, and 40% of the strongest edges.

#### Absolute thresholding.

Rather than selecting a threshold in terms of desired network density, an alternative approach is to only retain edges that have a minimum chosen strength, irrespective of how many such edges would survive in any given network. However, once again there is no gold standard to determine what value of strength would represent an appropriate threshold, leaving the choice largely arbitrary—even more so because different ways of estimating functional connectivity (e.g., Pearson correlation and mutual information) may give results in very different value ranges. Here, we considered absolute threshold values of 0.1, 0.3, or 0.5 (for Pearson correlation, only positive-value edges were considered).

#### Efficiency cost optimization.

Efficiency cost optimization (ECO) is a filtering scheme designed to optimize the trade-off, within a given network, between the network’s overall efficiency (sum of global and average local efficiency) and its wiring cost (number of edges; De Vico Fallani et al., 2017). Thus, it seeks the threshold density *ρ* that maximizes the function *J*, defined as follows:

$$J=\frac{E_{g}+E_{l}}{\rho },$$ (2)

with *E*_*g*_ and *E*_*l*_ being the global and mean local efficiency of the network, respectively. Empirical results have demonstrated, across multiple datasets and imaging modalities (MRI, EEG), that this filtering scheme is especially effective at discriminating between graph topologies, producing sparse graphs while still preserving their structure (De Vico Fallani et al., 2017). Analytic and empirical results also demonstrate that the required density to optimize the trade-off can be determined a priori once the number of nodes is known, and corresponds to enforcing an average node degree approximately equal to 3 (De Vico Fallani et al., 2017). Here, we thus obtained ECO-thresholded graphs by setting a proportional threshold such that the average node degree would be 3.

Because of the very high sparseness of ECO-derived networks as network size grows, we also wanted to ensure that any results we obtained were not merely driven by the network being highly fragmented. To this end, we also used an alternative version of ECO, termed MST-ECO. Like ECO, MST-ECO also imposes an average degree of 3 on the filtered graph, but ensures that the network’s minimum spanning tree is included among the selected edges—thereby producing a fully connected graph.

#### Orthogonal minimum spanning trees.

Similarly to ECO, the OMST approach (Dimitriadis, Antonakakis, et al., 2017; Dimitriadis, Salis, et al., 2017) also focuses on optimizing a function of the efficiency and cost of the network, with the added criterion of ensuring that the network is fully connected. Specifically, the method involves three steps: (a) identifying the minimum spanning tree (MST) of the network, that is, the minimum set of edges that constitute a fully connected graph; (b) removing the corresponding edges from the network; (c) repeating steps (a) and (b) until optimization of the following global cost efficiency function. The function is defined as *E*_*g*_ − Cost, and cost corresponds to the ratio of the total weight of the selected edges, over multiple iterations of OMST, divided by the total strength of the original fully weighted graph.

The final filtered network is constructed by combining all the MSTs that have been removed from the original dense network. The approach is data driven, as the trade-off is optimized for each network individually. Previous work has shown that when applied to brain networks derived from EEG and fMRI, this procedure can produce plausibly sparse graphs whose recognition accuracy and reliability outperform several alternative thresholding schemes (Dimitriadis, Salis, et al., 2017). This approach also produces sparse networks without imposing an a priori level across all subjects, thereby allowing for intersubject variability.

Note that although MST-ECO also incorporates one of the network’s minimum spanning trees (namely, the first MST that would be identified in the process of constructing the OMST-filtered network), any additional edges that MST-ECO selects to reach its target mean degree of 3 are based on strength alone; in contrast, OMST is obtained by combining not just one, but multiple nonoverlapping minimum spanning trees, each of which may select edges that are not among the strongest in the network.

#### Structural density matching.

Thresholding based on a fixed value or range of density or node degree may be more or less biologically meaningful, depending on the parcellation scheme used—because networks derived from more fine-grained parcellations of the same data (i.e., having higher numbers of nodes) may be expected to result in lower density. Two extreme cases will illustrate this notion. At one extreme, each neuron only has synapses with up to a few thousands of other neurons, out of approximately 86 billion—a vanishingly sparse network. Conversely, if considering each brain hemisphere as a single node, then the resulting (minimal) network will be fully connected. Thus, a 10% fixed density threshold (one of the most common choices in the literature) may be well below or well above the density of the corresponding network of white matter fibers between brain regions (estimated, for example, from diffusion imaging), depending on the size of the parcellation used.

To address this issue, we propose a three-step filtering method, which we call structural density matching (SDM). The idea behind SDM is simple. Since the main rationale for filtering FC networks is that brains have anatomically sparse connectivity, then it should be possible to threshold FC so as to match our best estimate of the density of structural connectivity (here understood as the network of white matter fibers connecting different regions). For a given parcellation scheme, the first step of SDM is to obtain the corresponding structural connectome. In humans, in vivo structural connectivity can be quantified by means of diffusion-weighted MRI; here, we rely on the group-averaged diffusion-weighted MRI data from the Human Connectome Project (Yeh et al., 2018), with two ROIs being considered as anatomically connected if there are white matter fibers between them. Then, the second step is to determine the “structural density” *s*, which is the density of the structural connectome obtained in the previous step (i.e., the proportion of existing edges, out of the total possible number of edges). Finally, the third step is to apply proportional thresholding to the functional connectivity data derived from the same parcellation scheme, with the target density (proportion of edges retained) being equal to *s*, the density of the structural connectome. That is, this procedure retains as many functional edges as there are edges in the DTI-derived structural connectome—thereby ensuring a biologically plausible level of sparsity, by construction. Importantly, note that this procedure does *not* amount to preserving exactly the same connections that are present in the structural connectome: only the same *number* of edges (as a proportion of the total). Which specific edges are retained for a given functional network will depend solely on their individual strength, which may or may not correspond to the presence of structural edges between the same regions (and in turn may be modulated by aspects such as tasks; Pappas, Craig, Menon, & Stamatakis, 2020).

SDM therefore views the number of anatomical connections as a biologically principled lower bound for the number of functional interactions: To be neurobiologically plausible, a functional network should not have fewer connections than the number of physical white matter connections in the brain (especially in the context of resting-state functional connectivity over the course of several minutes). Although the true number of functional connections is likely to be *higher* than the corresponding number of anatomical connections, because of the presence of polysynaptic indirect pathways between regions, making the threshold more lenient will also inevitably increase the likelihood of false positives. By fixing the density of the filtered functional network to be exactly *s*, SDM navigates this inevitable trade-off between false positives and false negatives by producing a network that is as sparse as possible (to keep the number of false positives to a minimum), but not so sparse as to become biologically implausible (by not having fewer functional edges than there are anatomical connections identifiable from DTI).

Effectively, SDM is a biologically principled way to choose which proportional threshold to adopt, which is otherwise largely arbitrary. Note that the threshold s selected by SDM may be higher or lower than popular alternatives such as 10% or 20% density, depending on the parcellation used (Table 3). By enforcing the same density across subjects, SDM suffers from the limitation of proportional thresholding mentioned above, that is, obscuring potentially meaningful differences in FC network density in between-groups designs (van den Heuvel et al., 2017). In the presence of DWI data for individual subjects, SDM can be easily modified to account for this potential confound, by using each individual’s own structural connectivity network density as the threshold s, which would preserve any density differences between different groups—thus overcoming one major criticism of current proportional thresholding approaches. However, DWI data are not always available, and here we therefore chose to use for all subjects the density of the HCP-1,021 group-average connectome, which can be used in any study since the underlying data are publicly available. This also represents a principled choice, in terms of being arguably the field’s current best estimate of the healthy human macroscale connectome.

**Table 3.** Density (percentage of total possible connections) of the human structural connectome, for each parcellation scheme included in the present study

Parcellation	Density (%)
AAL-90	19.2
Brainnetome-246	7.3
Glasser-414	2.8
Lausanne-129	10.9
Lausanne-234	5.9
Lausanne-463	2.3
Schaefer-116	14.7
Schaefer-232	7.4
Schaefer-454	3.6

Note that SDM is effectively predicated on the binarized structural network: By relying on only the total number of connections in the structural network (i.e., the network density), this method effectively ignores their individual weight—and therefore also the question of how to define it (e.g., whether normalization by distance or region area should be employed).

#### Random filtering.

Finally, to provide a baseline against which to compare the performance of the different filtering schemes considered up to this point, we also included an additional filtering scheme: randomly selecting 20% of connections in the connectivity matrix. For matrices obtained from Pearson correlation, if a negative connection was selected, its sign was turned to positive: This procedure was chosen to ensure that no connections would be a priori excluded from selection. Clearly, it is implausible that random sampling of an arbitrary proportion of connections should reveal the topological organization of the human brain. Therefore, unlike the other methods considered here—which are all intended to uncover the topological organization of the human brain—we should expect that this method will not yield networks with very representative topology.

### Binarization

We considered both weighted and unweighted networks in our analyses. Unweighted networks were constructed by setting all nonzero edges to unity. For functional connectivity network, this step was performed after filtering.

### Topological Distance as Portrait Divergence

The portrait divergence is based on an information-theoretic notion of distance (the Jensen-Shannon divergence) between graph invariants, encoding the distribution of shortest paths of the two networks, known as network portraits (Bagrow & Bollt, 2019). Specifically, the network portrait is a matrix *B* whose entry *B*_*lk*_, *l* = 0, 1, …, *d* (with *d* being the graph diameter), *k* = 0, 1, …, *N* − 1 is the number of nodes having *k* nodes at shortest-path distance *l*.

The number of nodes and edges, the degree distribution, the distribution of the next-nearest neighbors, and the number of shortest paths of length *l* are all encoded in *B*, which is a graph invariant; that is, it does not vary depending on how the graph is represented. Thus, comparing networks based on graph invariants is highly desirable, since it ensures that the comparison depends solely on the networks’ topology, without the confound of encoding format.

The portrait divergence distance between graphs G_1_ and G_2_ is then defined as follows. First, the probability *P*(*k*, *l*) (and similarly *Q*(*k*, *l*) for the second graph) of randomly choosing two nodes at distance *l* and, for one of the two nodes, to have *k* nodes at distance *l*, is computed as follows:

$$P(k,l)=P(k|l)P(l)=\frac{1}{N}B_{lk}\frac{1}{\sum_{c}n_{c}^{2}}\overset{N}{\underset{k^{\prime }=0}{\sum }}k^{\prime }B_{lk^{\prime }}$$ (3)

where *n*_*c*_ is the number of nodes in the connected component *c*. Then, the portrait divergence distance *D*(*G*_1_, *G*_2_)is defined using the Jensen-Shannon divergence:

$$D(G_{1},G_{2})=\frac{1}{2}KL(P∥M)+\frac{1}{2}KL(Q∥M),$$ (4)

where *M* = (*P* + *Q*)/2 is the mixture distribution of *P* and *Q*, and *KL*(⋅∥⋅) is the Kullback-Leibler divergence.

A chief advantage of this measure is that it can be applied to a wide variety of graphs: undirected or directed, binary or weighted (through binning of real-valued path lengths; here using 10 bins), connected or disconnected, and (most importantly for the present study) even if the two graphs differ in their number of nodes and edges—making it ideal for the purposes of the present analysis. Additionally, this measure simultaneously takes into account all scales of structure within networks, from local structure to motifs to large-scale connectivity patterns. Thus, it incorporates all aspects of network topology. Finally, the method is also computationally efficient (quadratic in the number of nodes), further widening the range of networks it can consider (Bagrow & Bollt, 2019).

## RESULTS

### Maximally Representative Parcellation

**Figure 1.**
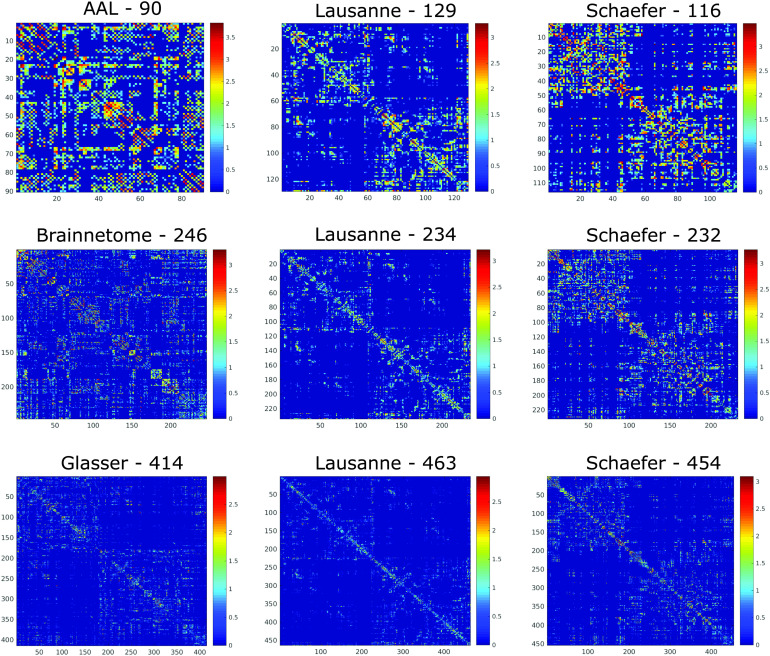
Structural connectome of the same individual according to different parcellations. Connections indicate the logarithm of the number of white matter streamlines between the corresponding regions (see Methods section), as identified by deterministic tractography.

**Figure 2.**
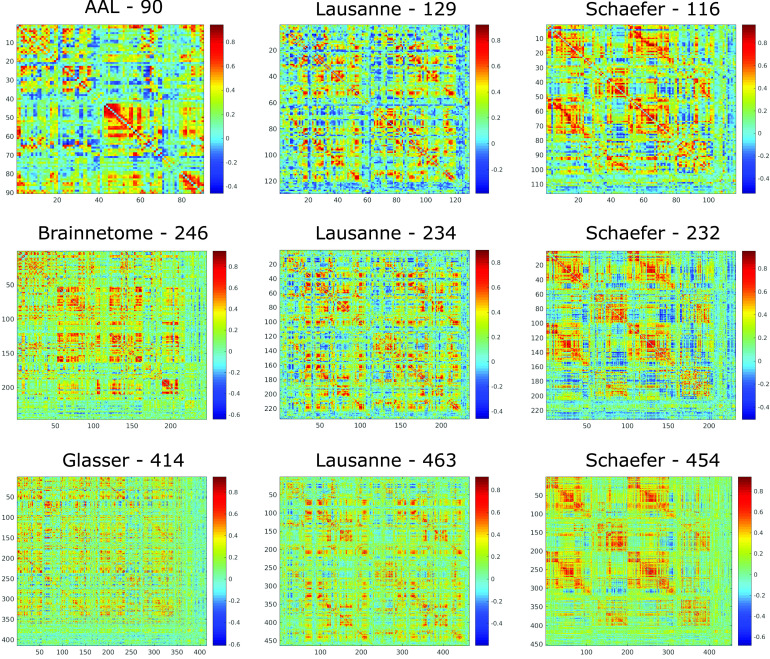
Functional connectome of the same individual according to different parcellations. Matrix entries indicate the Pearson correlation between the BOLD signal of the corresponding brain regions, obtained from resting-state fMRI of one representative HCP subject.

We first sought to determine which of the nine parcellation schemes considered here produces the most representative networks. We used both structural networks (Figure 1) and functional networks (Figure 2) for this analysis, and considered both weighted and binarized networks.

We began by computing the portrait divergence between the binarized structural connectivity networks, each obtained by parcellating a given subject’s structural connectome according to one of the nine parcellation schemes considered here. The average divergence of networks produced using each parcellation from all others (within the same subject) was then computed. We reasoned that the lower a parcellation’s average divergence from the others, the more this is a representative parcellation. Thus, our measure of representativeness for a given parcellation was defined as one minus its average divergence from all other parcellations.

**Figure 3.**
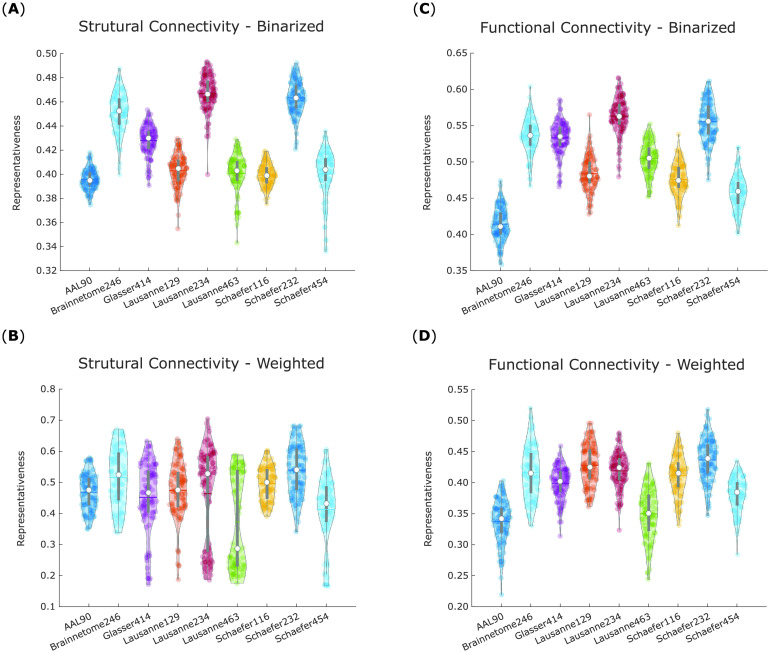
Scale-200 parcellations exhibit the highest representativeness for structural and functional networks. For each parcellation, violin plots show the distribution of its network representativeness (1 minus average portrait divergence with every other parcellation) based on the connectomes of 100 HCP subjects. (A) Binary networks from structural connectivity. (B) Weighted networks from structural connectivity. (C) Binary networks from functional connectivity. (D) Weighted networks from functional connectivity. White circle, mean; center line, median; box limits, upper and lower quartiles; whiskers, 1.5× interquartile range.

A within-subjects ANOVA (analysis of variance) was conducted to compare average representativeness of binarized structural networks across each of the nine parcellation schemes in our 100 subjects, revealing significant differences between them: *F*(8, 891) = 421.08, *p* < 0.001 (Figure 3A).

Likewise, significant differences were also obtained for weighted structural networks *F*(8, 891) = 23.13, *p* < 0.001 (Figure 3B). In this case, however, the differences between parcellations were much less pronounced, and the values of representativeness spanned a wider range (0.10 to 0.70, vis-à-vis 0.30 to 0.50 for binarized networks).

The same procedure was then repeated for binary and weighted networks obtained from functional connectivity (Pearson correlation). To obtain results comparable to structural networks, the functional networks were sparsified by retaining only the same proportion of edges as found in the corresponding HCP group-average structural connectome (which corresponds to applying the structural density matching procedure described in the Methods section).

These analyses again revealed significant differences in representativeness between parcellations, for both binary (*F*(8, 891) = 418.39, *p* < 0.001; Figure 3C) and weighted (*F*(8, 891) = 107.34, *p* < 0.001; Figure 3D) functional networks. Results from the binarized functional networks were remarkably similar to those from binarized structural networks, and a similar pattern was also followed by the weighted functional networks.

**Figure 4.**
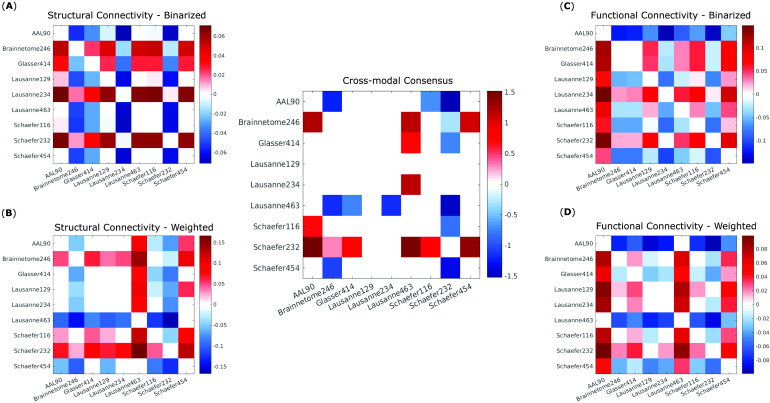
Significant differences in representativeness between individual parcellations. Each cell in a matrix indicates the difference (row > column) between the means of the corresponding distributions plotted as violins in Figure 3. Names of the parcellations are indicated in the corresponding row and column. Nonwhite cells indicate a statistically significant difference (paired-samples *t* test, Bonferroni-corrected). (A) Binary networks from structural connectivity. (B) Weighted networks from structural connectivity. (C) Binary networks from functional connectivity. (D) Weighted networks from functional connectivity. Central panel: significant differences that are present in all comparisons (A)–(D) in the same direction (all positive or all negative).

Follow-up paired-samples *t* tests (Bonferroni-corrected for multiple comparisons) were used to identify significant differences in representativeness between individual parcellations, for each of the four kinds of networks considered here: structural and functional, each binarized and weighted (Figure 4A–D). Based on these results, we could identify which parcellation schemes were significantly more or less representative than others, reliably across all four network types (Figure 4, central panel). In particular, the Schaefer-232 parcellation was at least as representative as any other parcellation considered here, and significantly more representative than most, regardless of network type. Brainnetome-246 also performed very well, being equally or more representative than any other parcellation except for Schaefer-232.

Bimodality in the distribution of representativeness was observed for weighted structural networks (Figure 3B). This phenomenon was observed for all scales of the Lausanne anatomical parcellation, and for each of the scale-400 atlases—with the two effects appearing to be to some extent cumulative, since the Lausanne-463 was the one with the most pronounced bimodality. This phenomenon can be attributed to some subjects having significantly denser structural networks than other subjects, leading to corresponding bimodality in the distribution of network density (Supplementary Figure 1 in the Supporting Information; all *p* < 0.001). While this difference does not seem to affect representativeness across parcellations for binary networks, it does for weighted networks, leading to lower representativeness for weighted structural networks obtained from anatomical or scale-400 parcellation.

### Regular-Random Representativeness

To complement the previous analysis of cross-parcellation representativeness, we also sought to determine how well each parcellation can distinguish between network topologies that are widely considered to lie at opposite ends of a continuum: random networks, and regular networks. To this end, structural and functional empirical networks obtained from each parcellation were turned into random or regular networks with the same weights distribution, as described in Muldoon, Bridgeford, and Bassett (2016). The representativeness between regular and random networks obtained from the same parcellation was then computed as 1 minus their portrait divergence, for both binary and weighted networks. We refer to this quantity as “regular-random representativeness,” to distinguish it from cross-parcellation representativeness between empirical networks (Figures 3 and 4). For the analysis of regular-random representativeness, low representativeness (i.e., high portrait divergence) is desirable, as it indicates that a given parcellation can correctly reflect the large theoretical difference between random and regular networks.

**Figure 5.**
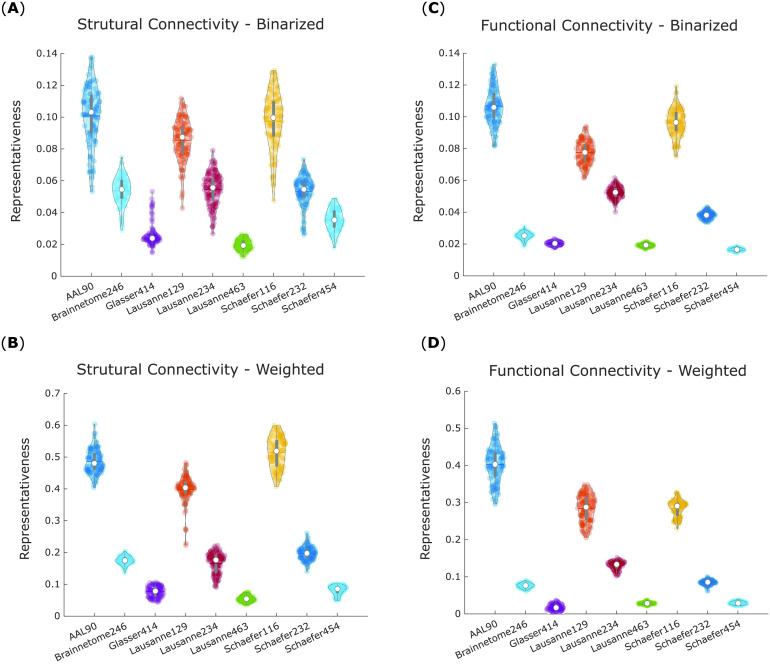
Regular-random representativeness inversely scales with parcellation size for both structural and functional networks. For each parcellation, violin plots show the distribution of network representativeness between regular and random networks (1 minus regular-random portrait divergence) obtained from it, for each of the 100 HCP subjects. (A) Binary networks from structural connectivity. (B) Weighted networks from structural connectivity. (C) Binary networks from functional connectivity. (D) Weighted networks from functional connectivity. White circle, mean; center line, median; box limits, upper and lower quartiles; whiskers, 1.5× interquartile range.

The different parcellations were compared in terms of regular-random representativeness by means of one-way within-subjects ANOVAs, revealing significant differences in mean regular-random representativeness, for binary (*F*(8, 891) = 650.07, *p* < 0.001; Figure 5A) and weighted (*F*(8, 891) = 3,552.63, *p* < 0.001; Figure 5B) structural networks, and also for binary (*F*(8, 891) = 3,857.59, *p* < 0.001; Figure 5C) and weighted (*F*(8, 891) = 3915.67, *p* < 0.001; Figure 5D) functional networks.

**Figure 6.**
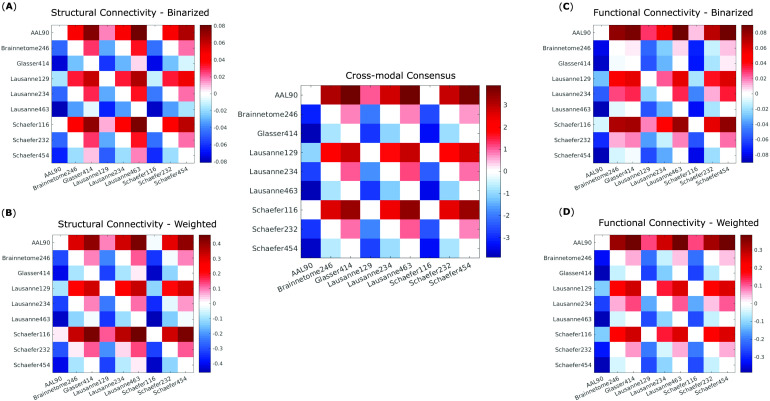
Significant differences in regular-random representativeness between parcellation scales. Each cell in a matrix indicates the difference (row > column) between the means of the corresponding distributions plotted as violins in Figure 5. Names of the parcellations are indicated in the corresponding row and column. Nonwhite cells indicate a statistically significant difference (paired-samples *t* test, Bonferroni-corrected). (A) Binary networks from structural connectivity. (B) Weighted networks from structural connectivity. (C) Binary networks from functional connectivity. (D) Weighted networks from functional connectivity. Central panel: significant differences that are present in all comparisons (A)–(D) in the same direction (all positive or all negative).

Further Bonferroni-corrected, repeated-measures *t* tests clearly show that, across imaging modalities and for both binary and weighted networks, regular-random representativeness appears to scale inversely with parcellation size: It is lower (i.e., random and regular networks have greater divergence) for parcellations with larger numbers of ROIs (Figures 5 and 6). Importantly, regular-random representativeness was substantially below the range of empirical cross-parcellation representativeness for all binary networks, as well as for weighted networks constructed from atlases in the 200- or 400-ROI scales (Figure 3). However, weighted networks constructed from scale-100 parcellations exhibited much higher regular-random representativeness, in the range of the empirical cross-parcellation representativeness obtained from the same parcellation. This behavior was observed for both functional and structural connectivity. In other words, regular and random weighted networks obtained from a given scale-100 parcellation are about as representative of each other, as real weighted networks obtained from that same scale-100 parcellation are representative of real networks obtained with different parcellations (whether structural or functional).

### Maximally Representative Filtering Scheme

We next focused on finding the filtering scheme for functional connectivity that produces the most representative networks. The purpose of edge filtering can be seen as highlighting the network’s “true” topology, which may be obscured by the presence of spurious connections. As our previous analysis demonstrates, the choice of parcellation also inevitably introduces topological differences, even when the underlying neuroimaging data are the same.

We reasoned that edge filtering can either reduce or further amplify the topological divergence introduced by the parcellation step. Thus, from the point of view of representativeness, an appropriate filtering scheme is one that minimizes the topological differences arising from the use of different parcellations, by converging on the same underlying network topology across different parcellations. In contrast, a poor filtering scheme will further exacerbate the idiosyncrasies introduced by node definition, so that the same neuroimaging data may end up as networks with very different organization. In other words, an appropriate filtering scheme should reveal the topology of the underlying brain network in a similar way, no matter how the brain was parcellated.

Therefore, we defined representativeness of a given filtering scheme as minimizing the divergence between FC networks produced from different parcellations of the same data (which we refer to as “cross-parcellation representativeness”). In other words, we sought the filtering scheme that minimizes the impact of one’s choice of parcellation. To ensure that our results were not dependent on the removal of negative correlations, we also computed FC based on mutual information, which is always nonnegative and can also account for nonlinear interactions (Supporting Information, Supplementary Figure 2). Both binarized and weighted functional networks were considered.

**Figure 7.**
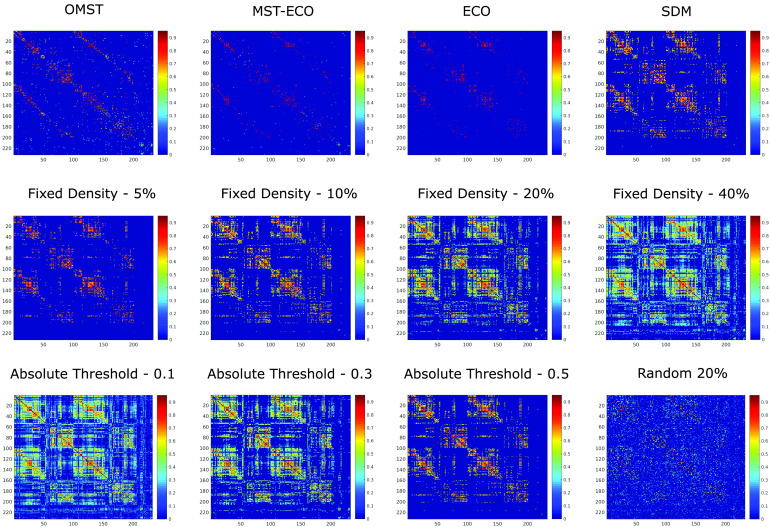
Distinct filtering schemes applied to the same functional connectome. The same functional connectome from one representative HCP subject (Schaefer-232, shown before filtering in Figure 2) is shown after filtering of its connections based on 12 different schemes, producing matrices of different sparsity. OMST, orthogonal minimum spanning trees. ECO, efficiency cost optimization. SDM, structural density matching. Fixed density indicates that a fixed percentage of the strongest weights are retained. Random 20% corresponds to preserving 20% of connections, chosen at random.

**Figure 8.**
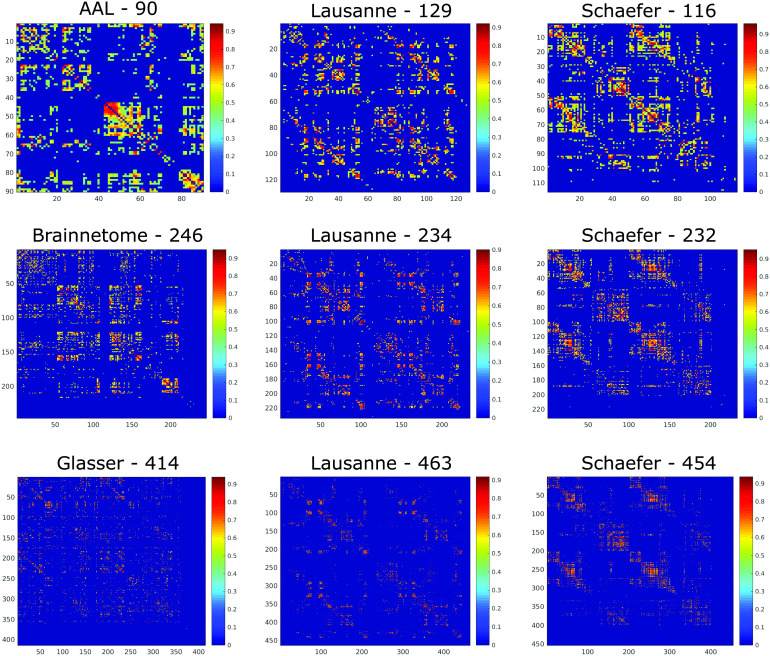
Same filtering scheme applied to different parcellations. The functional connectome from one representative HCP subject (based on Pearson correlation) is shown after filtering of its connections based on the structural density matching criterion, across the nine parcellation schemes used.

Thus, a total of (number of atlases) × (number of filtering schemes) × (number of functional connectivity measures) × (binary vs. weighted) = 9 × 12 × 2 × 2 = 432 distinct filtered functional connectivity matrices were obtained, from each subject’s BOLD signal data (Figures 7 and 8).

For each filtering scheme, we computed the portrait divergence between each of the nine functional networks derived from it (one per atlas) and every other one. As above, representativeness of a given filtering scheme was quantified as 1 minus the average divergence between that scheme’s resulting networks across parcellations, producing one value per filtering scheme, per subject.

**Figure 9.**
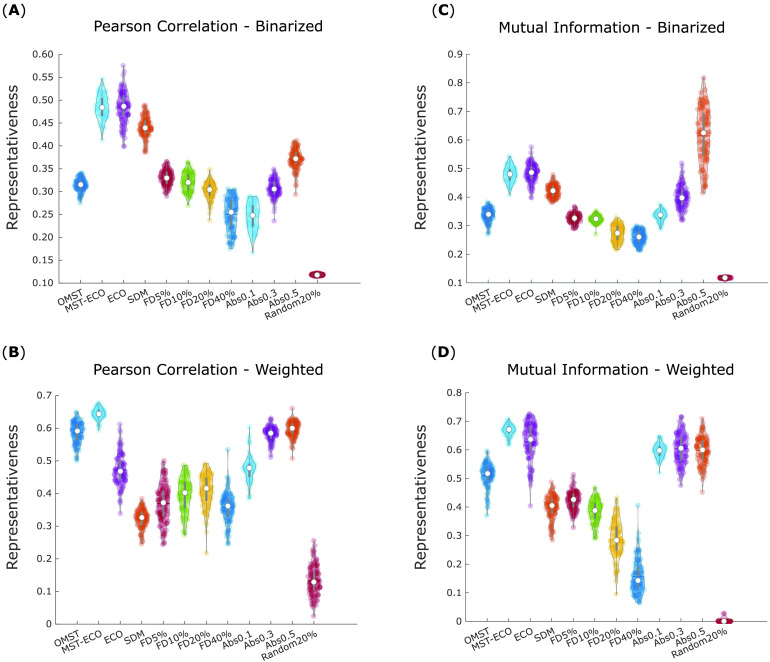
Representativeness of filtering schemes. For each filtering scheme, violin plots show the distribution of cross-parcellation representativeness for 100 HCP subjects. Each data point corresponds to the average representativeness of a given filtering scheme across parcellations (1 minus average portrait divergence between functional networks obtained from applying the same filtering scheme across all nine parcellations). (A) Binary networks based on Pearson correlation between BOLD signal time series. (B) Weighted networks based on Pearson correlation between BOLD signal time series. (C) Binary networks based on mutual information between BOLD signal time series. (D) Weighted networks based on mutual information between BOLD signal time series. White circle, mean; center line, median; box limits, upper and lower quartiles; whiskers, 1.5× interquartile range. OMST, orthogonal minimum spanning trees. ECO, efficiency cost optimization. SDM, structural density matching. FD, fixed density. Abs, absolute thresholding. Random 20% corresponds to preserving 20% of connections, chosen at random.

The cross-parcellation representativeness of different filtering schemes were compared by means of a one-way within-subjects ANOVA, revealing significant differences in mean representativeness across filtering schemes, for binary networks based on Pearson correlation (*F*(11, 1,188) = 2,070.40, *p* < 0.001; Figure 9A). Likewise, significant overall differences were also observed for weighted networks based on Pearson correlation (*F*(11, 1,188) = 1,283, *p* < 0.001; Figure 9B), and for binary (*F*(11, 1,188) = 1,239.84, *p* < 0.001; Figure 9C) and weighted (*F*(11, 1,188) = 2,098.05, *p* < 0.001; Figure 9D) networks based on mutual information.

**Figure 10.**
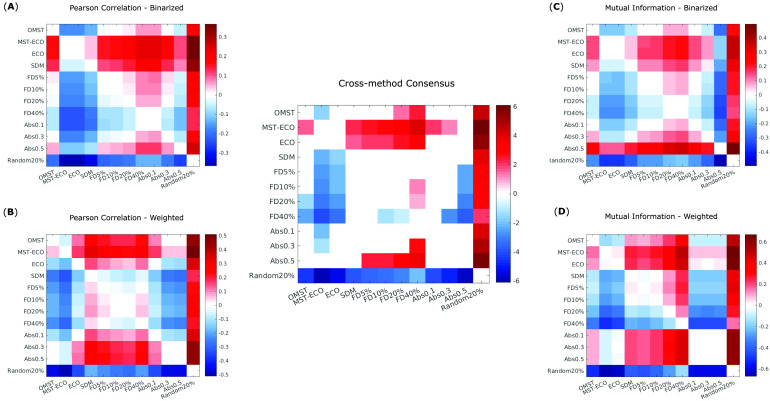
Significant differences in cross-parcellation representativeness of filtering schemes. Each cell in a matrix indicates the difference (row > column) between the means of the corresponding distributions plotted as violins in Figure 9. (A) Binary networks based on Pearson correlation between BOLD signal time series. (B) Weighted networks based on Pearson correlation between BOLD signal time series. (C) Binary networks based on mutual information between BOLD signal time series. (D) Weighted networks based on mutual information between BOLD signal time series. Central panel: significant differences that are present in all comparisons (A)–(D) in the same direction (all positive or all negative).

Further within-sample *t* tests (Bonferroni-corrected for multiple comparisons) identified ECO and especially its connected variant, MST-ECO, as being significantly more representative than all other filtering schemes, in almost all cases (Figure 10A–D and central panel). Absolute thresholding also performs well, especially with thresholds of 0.3 and 0.5. Absolute thresholding also results in much greater variability across subjects in terms of density of the resulting networks (Supporting Information, Supplementary Figure 3). Additionally, for MI the most stringent absolute threshold (edges stronger than 0.5) reliably results in some subjects having functional networks with density one order of magnitude smaller than the group-average structural connectome (Supporting Information, Supplementary Tables 1 and 2).

Additional insights emerge when considering binary or weighted networks. Namely, SDM produces binary networks that are highly representative across parcellations, whereas OMST is especially successful in weighted networks. Proportional thresholding reliably yielded networks with poor representativeness across parcellations, regardless of the chosen threshold. Nevertheless, across both weighted and binary networks, and for both MI and Pearson correlation, proportional thresholding still performed significantly better than filtering by randomly selecting 20% of edges. As expected, this method invariably exhibited by far the poorest performance, producing networks with significantly lower representativeness across parcellation than any other filtering scheme. This observation validates the notion that a poor choice of filtering scheme can exacerbate the differences introduced by the parcellation step, producing networks with extremely low representativeness across parcellations—as well as demonstrating the full extent of gains that can be obtained by choosing an appropriate filtering scheme.

## DISCUSSION

### How to Make a Representative Brain Network

Here, we reveal that significant differences arise between the representativeness of brain networks derived from different pipelines, in terms of both parcellation and filtering schemes adopted. When considering the choice of parcellation, networks obtained from the Schaefer-232 and Brainnetome-246 were the most representative, across both DTI and fMRI.

In particular, these parcellations appear to be especially appropriate for use with weighted networks. On the one hand, parcellations in the order of 100 nodes exhibit limited ability to discriminate between theoretically distinct weighted network topologies (random vs. regular). On the other hand, both scale-400 and anatomical parcellations result in bimodal distributions of representativeness for weighted networks, driven by differences in network density. As the only two parcellations that do not suffer from either of these potential problems, Schaefer-232 and Brainnetome-246 further emerge as clear recommendations.

The scale-200 parcellations occupy the middle ground between the three scales considered here, and so it stands to reason that their distance from the other two scales should be smaller than the distance between scale-100 and scale-400. However, our results are in line with the work of Arslan et al. (2018), who found lower gender classification accuracy for parcellations with fewer than 150 nodes, and few benefits from increasing the number of nodes beyond 350. Likewise, Messé (2020) recently argued for parcellations with 200–300 ROIs as a good compromise between the reliability of connectivity estimates, and the robustness of structural-functional correspondence.

In terms of filtering schemes for functional connectivity, we found a clear superiority of the efficiency cost optimization (ECO) scheme, especially in its non-disconnected variant (MST-ECO). This is in line with the rationale behind the development of this specific filtering scheme, which has been demonstrated to emphasize the intrinsic topology of a network (De Vico Fallani et al., 2017). Since our analysis of representativeness is based on minimizing the divergence between network topologies, it stands to reason that as the method most closely focusing on highlighting network topology, ECO should produce especially consistent results across parcellations.

Although some absolute thresholding also produced very representative networks across parcellations, the density of networks produced with this approach was highly variable with some subjects’ functional brain networks being an order of magnitude sparser than the average, and an order of magnitude sparser than the corresponding group-level structural connectivity. This raises concerns for the use of absolute thresholding because such stark variation in brain network density of healthy individuals appears implausible. Additionally, it seems hard to claim that the brain should have fewer functional connections (i.e., interactions between regions) than there are physical white matter connections between regions.

The number of anatomical connections may thus be seen as a biologically principled lower bound of the number of functional interactions. By using this number to decide how many connections to retain, structural density matching produced binary networks with significantly lower topological divergence than almost any other filtering method, regardless of how functional connectivity was quantified. Therefore, we believe that using SDM over ECO or stringent absolute thresholding may be warranted—in the context of binary networks, specifically—whenever those alternative filtering schemes would lead to networks with fewer functional connections than the corresponding number of anatomical connections.

Nevertheless, it is intriguing that SDM appeared to perform poorly in the context of weighted networks. This behavior may be because SDM considers only the number of structural connections when determining the threshold *s*, and disregards their individual strength. Future extensions of SDM that also take into account the strength of structural connections may provide improved performance for SDM on weighted networks.

Conversely, the OMST approach was especially successful for weighted networks, despite performing relatively poorly after binarization. Uniquely among the filtering schemes considered here (except for MST-ECO), OMST does not restrict itself to preserving only the strongest edges, and OMST-derived networks are likely to contain a number of edges that all other methods would discard as too weak. The importance of weak connections acting as “shortcuts” to improve efficiency in the brain has been increasingly recognized (Gallos, Makse, & Sigman, 2012; Gallos, Sigman, & Makse, 2012), and indeed OMST outperformed several other thresholding schemes in terms of both recognition accuracy and reliability of the resulting graph metrics (Dimitriadis, Salis, et al., 2017; Messaritaki et al., 2019).

### Limitations

This study also had a number of limitations that should be borne in mind when considering our recommendations. First, the space of all possible node definitions and filtering procedures is extremely vast, and therefore our sampling of it is not exhaustive: A wide variety of atlases exist, at multiple scales, and based on several different criteria—leading to combinatorial explosion (Arslan et al., 2018; Eickhoff et al., 2018).

Some investigators even refrain from using predefined atlases altogether, generating networks from individual voxels (Du et al., 2015), or from data-driven approaches such as independent components analysis (Kiviniemi et al., 2009; Smith et al., 2011). Although considering all possible node definition approaches would not have been feasible, we did endeavor to make our sampling systematic, by varying both the size (approximately 100, 200, and 400 nodes) and the parcellation scheme (based on neurobiological considerations, structural connectivity, and functional connectivity), thereby covering a number of the most common approaches—though at the cost of excluding the cerebellum.

Likewise, there exist a number of filtering schemes beyond those considered here: from thresholding based on statistical significance or regularization (Smith et al., 2011), to the soft thresholding approach of Schwarz and McGonigle (2011), or indeed no threshold at all, by employing algorithms that can deal with maximally dense, weighted, and even signed graphs (Rubinov & Sporns, 2011). Further work may also explore additional ways to define network edges, from the use of partial correlation (Smith et al., 2011) to directed connectivity methods, such as transfer entropy, dynamic causal modeling, and Granger causality, to networks derived from other neuroimaging modalities, such as MEG or EEG.

It also remains an open question how well our results would generalize to task-based functional data, given evidence that functional parcellations themselves vary across tasks (Salehi et al., 2020). It is also hotly debated whether preprocessing steps such as global signal regression have beneficial (Braun et al., 2012; Welton et al., 2015), deleterious (H. Cao et al., 2014), or negligible (Andellini et al., 2015; Du et al., 2015) effects on subsequent network construction.

Crucially, the definition of the structural connectome density on which SDM relies is itself subject to limitations. Diffusion MRI is only an indirect measure of in vivo structural connectivity; although correlated with tract-tracing results (Delettre et al., 2019; Donahue et al., 2016), dMRI tractography also suffers from false positives and difficulties resolving crossing fibers, and not all white matter connections may be correctly identified (Yeh et al., 2018). Tractography results may also depend on acquisition and diffusion-weighting protocols (Messaritaki et al., 2019). Furthermore, alternative methods for structural network reconstruction such as probabilistic tractography can themselves require the choice of a filtering scheme before binarization—highlighting the key dependence of SDM on deterministic tractography to produce inherently sparse structural networks. Since deterministic tractography requires a number of parameters to be selected, the associated limitations of this approach should be considered as well as the more general limitations of dMRI.

As a final note, we acknowledge that a hypothetical, highly accurate method for deriving networks from brain data (in the sense of being the closest to the ground truth) could theoretically produce networks that turn out to be topologically very different from the networks produced by current state-of-the-art methods. Though closest to the ground truth, this hypothetical best method would rank poorly on our criterion of representativeness. In other words, we acknowledge the general point that consensus is not guaranteed to correspond to the truth; indeed, history teaches that sometimes it is those with idiosyncratic convictions who are right, and the majority’s consensus wrong (think of Copernicus or Galileo). Nevertheless, in the current absence of such a ground truth for the brain’s network organization, we believe that representativeness among state-of-the-art methods such as those considered here may constitute a useful guiding principle.

We also emphasize that the representativeness of brain networks is only one of several factors that neuroscientists may wish to consider when deciding how to turn their neural data into networks. For instance, studies seeking how to best classify patients may opt to construct networks based on pipelines optimized for this goal—whereas longitudinal studies will likely benefit from the adoption of methods with high test-retest reliability. More generally, it remains a matter for future research to determine the relationship and trade-offs between representativeness, reliability, and empirical usefulness. Nevertheless, we believe that network representativeness can provide additional guidance in the choice of network construction pipelines.

### Conclusion

Overall, our proposed criterion of representativeness based on network portrait divergence identifies specific node definition and filtering procedures that neuroscientists can follow, in order to derive maximally representative brain networks from their neuroimaging data. To summarize, parcellations in the order of 200 brain regions—and especially the augmented Schaefer-232 and Brainnetome-246 parcellations—yield the most topologically representative brain networks across modalities and network types. Its consistently superior performance across edge definitions and network types makes efficiency cost optimization the method of choice for edge filtering, based on representativeness alone. Alternatively, for binary networks structural density matching may represent a good compromise between representativeness and neurobiological plausibility (i.e., producing a network that is neither exceedingly dense nor implausibly sparse), whereas OMST may represent a suitable, less sparse alternative to ECO for use in weighted networks.

## ACKNOWLEDGMENTS

The authors are grateful to Helena Gellersen for providing the motivation that inspired this work, and to members of the Cognition and Consciousness Imaging Group for helpful discussion.

## DATA AND CODE AVAILABILITY

The HCP DWI data in SRC format are available online (http://brain.labsolver.org/diffusion-mri-data/hcp-dmri-data). The HCP fMRI data are available online (https://www.humanconnectome.org/study/hcp-young-adult/data-releases). The HCP DTI population-averaged template is freely available online (http://brain.labsolver.org/diffusion-mri-templates/hcp-842-hcp-1021).

The CONN toolbox is freely available online (http://www.nitrc.org/projects/conn). Python code for the portrait divergence is freely available online (https://github.com/bagrow/network-portrait-divergence). MATLAB code for the orthogonal minimum spanning tree thresholding is freely available online (https://github.com/stdimitr/topological_filtering_networks). The Brain Connectivity Toolbox code used for graph-theoretical analyses is freely available online (https://sites.google.com/site/bctnet/).

## SUPPORTING INFORMATION

Supporting information for this article is available at https://doi.org/10.1162/netn_a_00170.

## AUTHOR CONTRIBUTIONS

Andrea I. Luppi: Conceptualization; Data curation; Formal analysis; Investigation; Methodology; Validation; Visualization; Writing - Original Draft. Emmanuel A. Stamatakis: Conceptualization; Funding acquisition; Project administration; Resources; Supervision; Writing - Review & Editing.

## FUNDING INFORMATION

Andrea I. Luppi, Gates Cambridge Trust (http://dx.doi.org/10.13039/501100005370). Emmanuel A. Stamatakis, Queens’ College Cambridge, Stephen Erskine Fellowship. Computing infrastructure at the Wolfson Brain Imaging Centre (WBIC-HPHI), Medical Research Council (http://dx.doi.org/10.13039/501100000265), Award ID: MR/M009041/1. Data were provided by the Human Connectome Project, WU-Minn Consortium (principal investigators: David Van Essen and Kamil Ugurbil), funded by the McDonnell Center for Systems Neuroscience at Washington University, and the 16 NIH Institutes and Centers that support the NIH Blueprint for Neuroscience Research (http://dx.doi.org/10.13039/100000135), Award ID: 1U54MH091657.

## TECHNICAL TERMS

Structural connectivity: The network of physical connections between macroscale neuronal ensembles (brain regions), alternatively referred to as anatomical connectivity or the connectome.
Functional connectivity: The network of interactions between brain regions, in terms of statistical association between their activity over time.
Edge filtering: The procedure of choosing which connections (edges) to retain in a network.
Graph theory: The mathematical study of networks.
Information theory: The formal study of storage and transmission of information.
Parcellation: Division of the brain’s gray matter into nonoverlapping macroscopic ensembles (called parcels or regions of interest) based on predetermined criteria.
Diffusion MRI (dMRI): MRI sequence estimating the orientation of diffusion of water molecules in tissue, producing diffusion-weighted images (DWIs).
Network topology: The way that elements in a network (nodes and edges) are organized.
Deterministic tractography: Technique to determine the presence or absence of white matter streamlines between brain regions based on diffusion-weighted images.
