# Supplementary material for: Combining network topology and information theory to construct representative brain networks
Source: Netw Neurosci. 2021 Feb 1;5(1):96–124. doi: 10.1162/netn_a_00170 (PMC7935031; doi:10.1162/netn_a_00170)
Supplement: Supplementary file 1 [file netn-05-96-s001.pdf]

Luppi, A. I. & Stamatakis, E. A. (2021). Supporting information for "Combining Network Topology and Information Theory to Construct Representative Brain Networks." *Network Neuroscience*., 5(1), 96–124. https://doi.org/10.1162/netn_a_00170

Combining Network Topology and Information Theory to Construct Representative Brain Networks

Andrea I. Luppi[a,b], Emmanuel A. Stamatakis[a,b]

[a]Division of Anaesthesia, School of Clinical Medicine, University of Cambridge, United Kingdom
[b]Department of Clinical Neurosciences, University of Cambridge, Cambridge, United Kingdom

Supplementary Information

Supplementary Figures

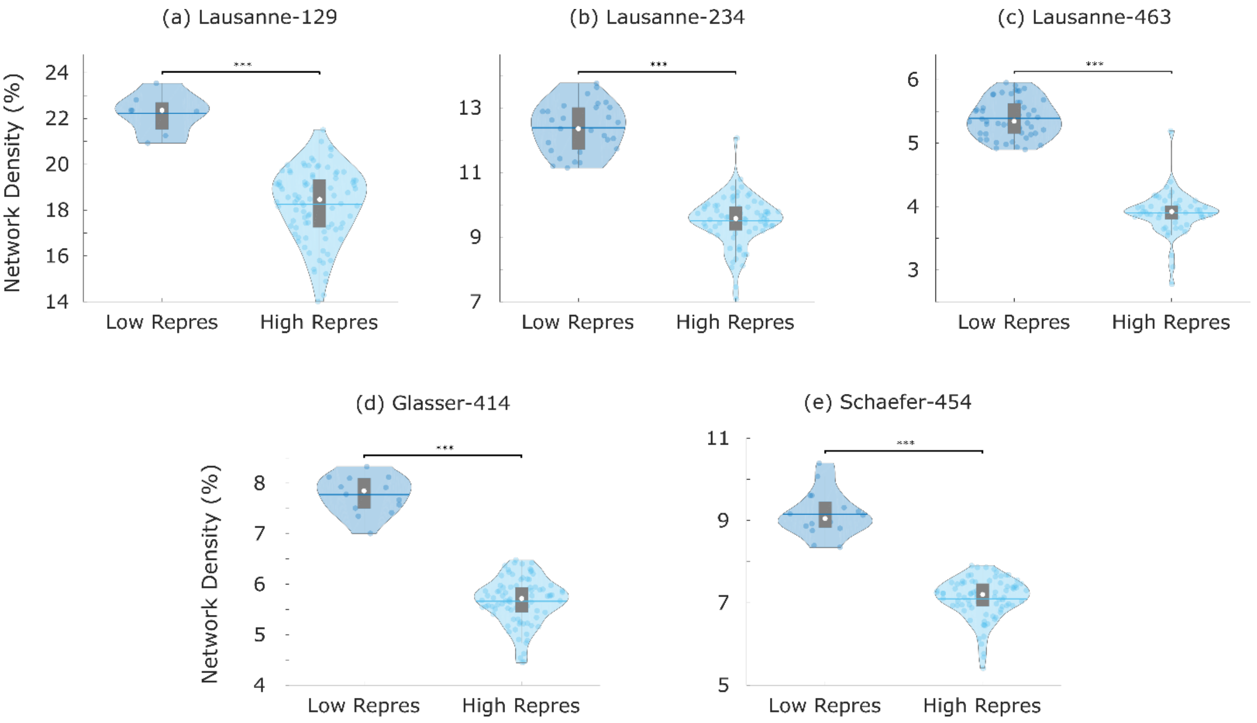

Supplementary Figure 1. Lower-representativeness of weighted structural networks corresponds to significantly higher network density. For weighted structural networks exhibiting bimodality in the distribution of representativeness (Figure 3b), subjects were divided into higher-representativeness and lower-representativeness groups, based on which of the two modes of the distribution they clustered around. For each parcellation (a-c, Lausanne-129, 234 and 463; d, Glasser-414; e, Schaefer-454), subjects with lower representativeness had significantly higher network density. Repres, representativeness. White circle, mean; center line, median; box limits, upper and lower quartiles; whiskers, 1.5x interquartile range. *** $p < 0.001$.

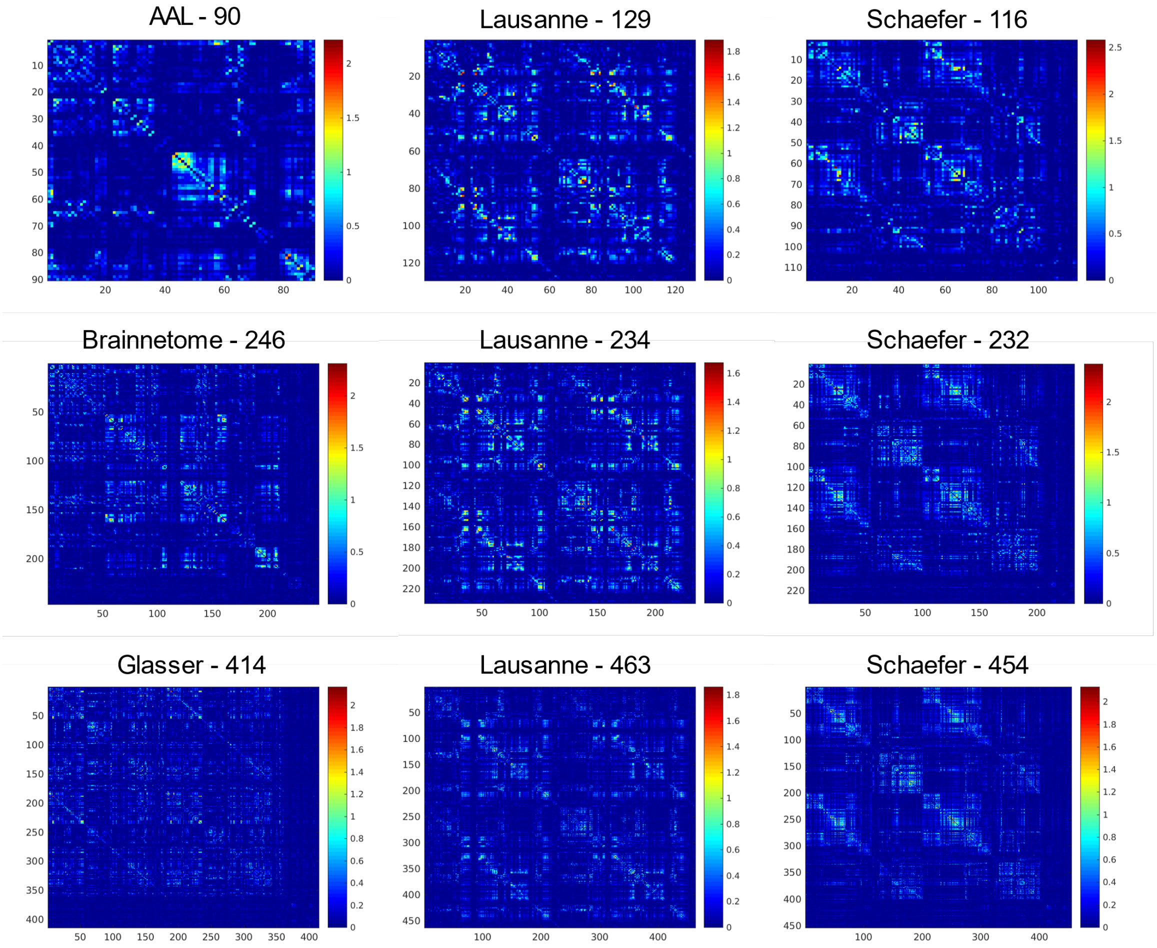

Supplementary Figure 2. Functional connectome of the same individual according to different parcellations, based on mutual information. Matrix entries indicate the mutual information between the BOLD signal of the corresponding brain regions, obtained from resting-state fMRI of one representative HCP subject. Matrices are here shown before normalisation in the range 0-1, and therefore values may exceed unity.

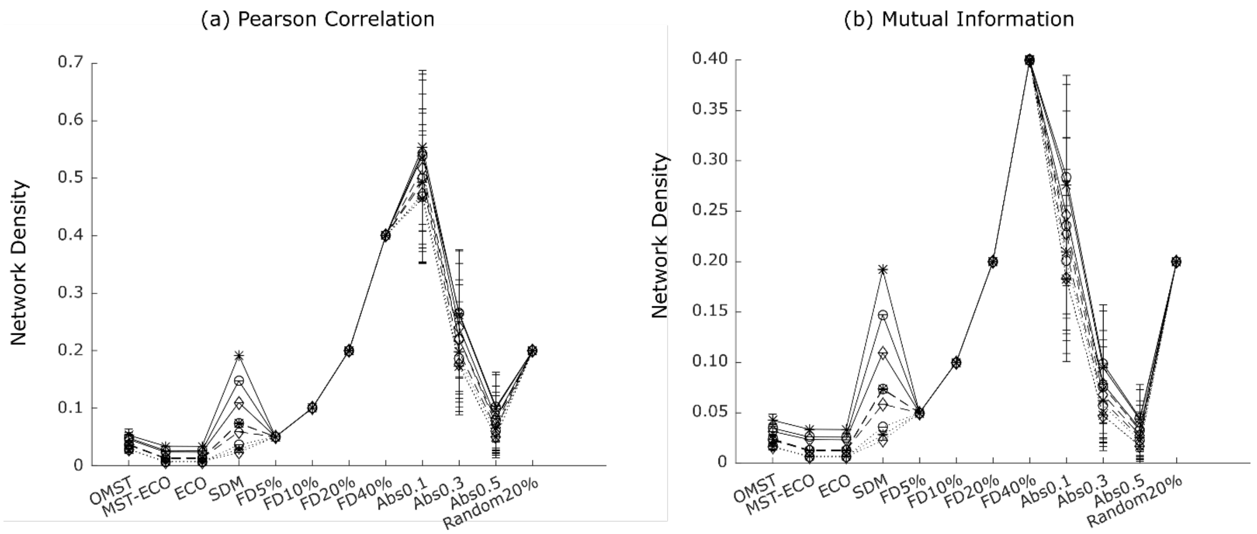

Supplementary Figure 3. Density of functional networks (mean and standard deviation) based on Pearson correlation (a) and mutual information (b) across different filtering schemes, and for different parcellations. OMST, Orthogonal Minimum Spanning Trees. ECO, Efficiency-Cost Optimisation. SDM, Structural Density Matching. FD, Fixed Density. Abs, absolute thresholding. Solid lines: scale-100 parcellations. Dashed lines: scale-200 parcellations. Dotted lines: scale-400 parcellations. Diamond: anatomical parcellations (Lausanne). Circle: functional parcellations (Schaefer). Asterisk: mixed parcellations (AAL, Brainnetome, Glasser).

Supplementary Tables

Supplementary Table 1. Minimum network density (as a percentage of total possible edges) across parcellations and filtering schemes, for networks of functional connectivity based on Pearson correlation.

	OMST	ECO	ECO-MST	SDM	FD-5%	FD-10%	FD-20%	FD-40%	Abs0.1	Abs0.3	Abs0.5	Rand 20%
**AAL-90**	4.4	3.4	3.3	19.2	5.0	10.0	20.0	40.0	32.2	10.8	3.2	20.0
**Brainnetome-246**	2.4	1.2	1.2	7.3	5.0	10.0	20.0	40.0	29.9	7.4	2.1	20.0
**Glasser-414**	1.9	0.7	0.7	2.8	5.0	10.0	20.0	40.0	28.5	6.5	1.5	20.0
**Lausanne-129**	3.1	2.3	2.3	10.9	5.0	10.0	20.0	40.0	32.8	8.9	2.5	20.0
**Lausanne-234**	2.6	1.3	1.3	5.9	5.0	10.0	20.0	40.0	31.6	8.3	2.2	20.0
**Lausanne-463**	2.2	0.6	0.6	2.3	5.0	10.0	20.0	40.0	29.3	6.6	1.3	20.0
**Schaefer-116**	3.4	2.6	2.6	14.7	5.0	10.0	20.0	40.0	32.2	11.1	3.0	20.0
**Schaefer-232**	2.6	1.3	1.3	7.4	5.0	10.0	20.0	40.0	30.7	8.6	2.5	20.0

**Schaefer454**	2.2	0.7	0.7	3.6	5.0	10.0	20.0	40.0	29.2	7.1	1.9	20.0

Supplementary Table 2. Minimum network density (as a percentage of total possible edges) across parcellations and filtering schemes, for networks of functional connectivity based on mutual information.

	OMST	ECO	ECO-MST	SDM	FD-5%	FD-10%	FD-20%	FD-40%	Abs0.1	Abs0.3	Abs0.5	Rand20%
**AAL-90**	2.2	3.4	3.3	19.2	5.0	10.0	20.0	40.0	11.2	2.9	1.2	20.0
**Brainnetome-246**	1.6	1.2	1.2	7.3	5.0	10.0	20.0	40.0	8.0	2.0	0.7	20.0
**Glasser-414**	1.4	0.7	0.7	2.8	5.0	10.0	20.0	40.0	7.0	1.4	0.4	20.0
**Lausanne-129**	3.1	2.3	2.3	10.9	5.0	10.0	20.0	40.0	9.5	2.3	0.8	20.0
**Lausanne-234**	1.7	1.3	1.3	5.9	5.0	10.0	20.0	40.0	8.9	2.0	0.6	20.0
**Lausanne-463**	0.9	0.6	0.6	2.3	5.0	10.0	20.0	40.0	7.1	1.2	0.4	20.0
**Schaefer-116**	1.7	2.6	2.6	14.7	5.0	10.0	20.0	40.0	12.4	2.8	1.2	20.0
**Schaefer-232**	1.7	1.3	1.3	7.4	5.0	10.0	20.0	40.0	9.5	2.4	0.9	20.0
**Schaefer454**	1.3	0.7	0.7	3.6	5.0	10.0	20.0	40.0	8.0	1.8	0.5	20.0
